# Demethylating therapy increases cytotoxicity of CD44v6 CAR-T cells against acute myeloid leukemia

**DOI:** 10.3389/fimmu.2023.1145441

**Published:** 2023-04-27

**Authors:** Ling Tang, Yingjie Kong, Haobing Wang, Ping Zou, Ting Sun, Ying Liu, Juan Zhang, Na Jin, Hanwen Mao, Xiaojian Zhu, Jue Wang, Fankai Meng, Yong You

**Affiliations:** ^1^ Institute of Hematology, Union Hospital, Tongji Medical College, Huazhong University of Science and Technology, Wuhan, China; ^2^ Department of Hematology, Tongji Hospital, Tongji Medical College, Huazhong University of Science and Technology, Wuhan, China; ^3^ Department of R&D, Wuhan Biological Sample Bank Co., Ltd., Wuhan, China; ^4^ Department of Oncology, The First People’s Hospital of Jiangxia District, Wuhan City and Union Jiangnan Hospital, Huazhong University of Science and Technology, Wuhan, China; ^5^ Oncology Department, Wuhan Dongxihu District People’s Hospital, Wuhan, China

**Keywords:** decitabine, azacitidine, CD44v6 chimeric antigen receptor-T cells, acute myeloid leukemia, demethylating therapy

## Abstract

**Background:**

CD44v6 chimeric antigen receptor T (CD44v6 CAR-T) cells demonstrate strong anti-tumor ability and safety in acute myeloid leukemia (AML). However, the expression of CD44v6 on T cells leads to transient fratricide and exhaustion of CD44v6 CAR-T cells, which affect the application of CD44v6 CAR-T. The exhaustion and function of T cells and CD44v6 expression of AML cells are associated with DNA methylation. Hypomethylating agents (HAMs) decitabine (Dec) and azacitidine (Aza) have been widely used to treat AML. Therefore, there may be synergy between CD44v6 CAR-T cells and HAMs in the treatment of AML.

**Methods:**

CD44v6 CAR-T cells pretreated with Dec or Aza were co-cultured with CD44v6+ AML cells. Dec or aza pretreated AML cells were co-cultured with CD44v6 CAR-T cells. The cytotoxicity, exhaustion, differentiation and transduction efficiency of CAR-T cells, and CD44v6 expression and apoptosis in AML cells were detected by flow cytometry. The subcutaneous tumor models were used to evaluate the anti-tumor effect of CD44v6 CAR-T cells combined with Dec *in vivo*. The effects of Dec or Aza on gene expression profile of CD44v6 CAR-T cells were analyzed by RNA-seq.

**Results:**

Our results revealed that Dec and Aza improved the function of CD44v6 CAR-T cells through increasing the absolute output of CAR+ cells and persistence, promoting activation and memory phenotype of CD44v6 CAR-T cells, and Dec had a more pronounced effect. Dec and Aza promoted the apoptosis of AML cells, particularly with DNA methyltransferase 3A (DNMT3A) mutation. Dec and Aza also enhanced the CD44v6 CAR-T response to AML by upregulating CD44v6 expression of AML cells regardless of FMS-like tyrosine kinase 3 (FLT3) or DNMT3A mutations. The combination of Dec or Aza pretreated CD44v6 CAR-T with pretreated AML cells demonstrated the most potent anti-tumor ability against AML.

**Conclusion:**

Dec or Aza in combination with CD44v6 CAR-T cells is a promising combination therapy for AML patients.

## Highlights

1. Decitabine and azacitidine pretreatment of CD44v6 CAR-T enhanced the anti-tumor ability of CD44v6 CAR-T against AML.
2. Decitabine and azacitidine enhanced the CD44v6 CAR-T response to AML by upregulating CD44v6 expression of AML cells.
3. Decitabine promoted stronger cytotoxicity of CD44v6 CAR-T cells towards AML compared to azacitidine.

## Introduction

Although patients with acute myeloid leukemia (AML) can achieve complete remission after standardized induction chemotherapy or allogeneic hematopoieticstem cell transplantation, most patients eventually relapse and have poor outcomes (1, 2).

Monoclonal antibodies, such as those targeting FMS-like tyrosine kinase 3 (FLT3), have increased remission rates in AML patients, while relapse and acquired drug resistance remain a major challenge (3, 4). In the past few years, several chimeric antigen receptor T (CAR-T) cells therapies have been explored to treat AML, including CD33, CD123, FLT3, and CLL-1 CAR-Ts (5–8). However, these antigens are also found in hematopoietic stem cells and progenitor cells, restricting their development.

CD44 isoform variant 6 (CD44v6) was highly expressed in AML blasts in contrast to hematopoietic stem cells and progenitor cells, making CD44v6 an optimal target for AML (9–11). Our previous study revealed that FLT3 or DNA methyltransferase 3A (DNMT3A) mutant AML cells overexpressed CD44v6 and CD44v6 CAR-T cells are significantly effective against them (9). CAR-T cells may become dysfunctional, expand and persist poorly due to inherent T-cell deficiencies (12, 13). T cells expressing CD44v6 resulted in transient fratricide and exhaustion of CD44v6 CAR-T (9). Therefore, it is necessary to explore effective strategies for improving CD44v6 CAR-T applications.

Our previous study revealed that FLT3 or DNMT3A mutations decreased CD44 promoter methylation, leading to CD44v6 overexpression (9), suggesting that CD44v6 expression was related to DNA methylation levels. Other studies have also shown that hypomethylating agents (HMAs) decitabine (Dec) or azacitidine (Aza) enhance immunotherapy by upregulating the expression of tumor-associated antigen in cancer cells (14–18). In recent years, Dec and Aza have been widely used to treat refractory AML patients and significantly improve the survival of patients (19–21). Therefore, we hypothesized that the up-regulation of CD44v6 expression by Dec or Aza may enhance the cytotoxicity of CD44v6 CAR-T against tumor cells. Furthermore, DNMT3a-mediated DNA methylation induces T cell exhaustion (22), as well as affecting T cell differentiation and activity (23–25). In T-cell studies with Dec or Aza, DNA methylation-induced exhaustion was reversed and the anti-tumor response was enhanced (26). These data suggest a potential synergy between CD44v6 CAR-T therapy and HMAs, both of which have shown significant efficacy in AML.

Hence, we proposed that CD44v6 CAR-T in combination with HMAs is a more prospective therapy for AML. Here, we evaluated the influence of Dec and Aza on CD44v6 CAR-T, AML cells, and responses of CD44v6 CAR-T cells to AML. We additionally compared the difference in the effects of Dec and Aza in this combined treatment.

## Materials and methods

### Cell culture

The China Center for Type Culture Collection provided human AML cell lines MV4-11 and SKM-1. The DNMT3A R882H (CGC>CAC) mutant clones of SKM-1 cells were constructed using lentiviral transduction, named SKM-1-DNMT3A-SC2 and SKM-1-DNMT3A-SC3, respectively. We used RPMI 1640 (Gibco) with 10% fetal bovine serum (FBS, Gibco) to culture AML cells and X-VIVO 15 (Lonza, USA) with 5% FBS and 200 U/mL recombinant human IL-2 (PeproTech, USA) to culture T cells.

### Construction of CD44v6 CAR-T

CD3/CD28 beads (Miltenyi Biotech, Cat# 130-091-441) were used to activate CD3+ T cells isolated from peripheral blood of healthy donors, from whom the informed consent was obtained. After two days, CAR lentivirus carrying anti-CD44v6-scFv-CD8-4-1BB-CD3ζ was added to activated T cells cultured in a density of 2×10^6^/mL. The multiplicity of infection was 3. Thirty min were spent centrifuging the mixture at 800 × *g.* After 24 h, transduction was terminated by replacing the medium with fresh medium.

### Flow cytometry analysis

The CAR-expressing T cells were identified by incubating with Biotinylated Protein L (GeneScript, Cat# M00097) at 4°C for 45 min and then washing them off before incubating with streptavidin-FITC (BD Biosciences, Cat# 554060) for 15 min in the dark. To analyze apoptosis, Annexin V-FITC/PI kit (BD Biosciences, Cat# 556547) was used to detect Dec (Selleck, NSC 127716) or Aza (Sigma, A2385) treated CAR-T cells or tumor cells. Assays involving cells surface antigens were performed in the dark at 4°C for 30 min with the corresponding antibodies. Antibodies (BD Biosciences) TIM3-PE (Cat# 563422) and CD69-BV421 (Cat# 562884) were applied to test the exhaustion and activation of CD44v6 CAR-T. CD4-BB700 (Cat# 566392), CD8-PE-cy7 (Cat# 557746), CCR7-Alexa 647 (Cat#557734), CD45RA-PE (Cat#555489) or CD45RA-BV421 (Cat# 562885) were utilized for detecting the phenotype of non-transduced T (NT) or CAR-T cells. CD44v6-PE (Cat# 566803) was applied to test the CD44v6 expression in AML cells. The samples were all detected by BD LSRFortessa X-20 flow cytometry, and these data were analyzed with FlowJo_V10.4 software.

### Number of CAR+ cells

On day 6 after transfection, CD44v6 CAR-T (1×10^5^) cells were treated with Dec (0.05, 0.1 μM) or Aza (0.5, 1 μM) for 6 days, the number of CD44v6 CAR-T cells in the untreated and drug-treated groups was counted under the microscope. We calculated the absolute number of CAR+ based on the counts of CD44v6 CAR-T cells and the percentage of CAR+ detected by flow cytometry.

### Cytotoxicity analysis

A 10:1 ratio of effector cells: target cells (E: T) was used in co-culturing NT or CAR-T cells pretreated with Dec (0.05, 0.1 μM) and Aza (0.5, 1 μM) for 6 days with CFSE-labeled (BD Biosciences, Cat#565082) CD44v6^+^ MV4-11 cells for 24 h. The same E: T ratio and co-culture time were used in incubating Dec 1 μM or Aza 1 μM pretreated AML cells with CD44v6 CAR-T cells. Dead cells were detected *via* flow cytometry using Fixable Viability Kit (Biolegend, Cat#423102) staining. The percentage of dead CFSE-positive cells was used to evaluate the cytotoxicity.

### Degranulation assay

A 1: 1 ratio was used in co-culturing NT or CAR-T cells pretreated with Dec (0.05, 0.1μM) or Aza (0.5, 1μM) with MV4-11 cells for 16 h. Meanwhile CD107a-BV421 (BD Biosciences, Cat#562623) and protein transport inhibitor (BD Biosciences, Cat#554724) were added to the culture medium. After cells collection, Antibodies CD3-APC/Cy7 (Biolegend, Cat#300318), CD4-BB700 (BD Biosciences, Cat# 566392), and CD8-BB515 (BD Biosciences, Cat# 564526) were applied to stain the cells.

### Cytokine release analysis

CD44v6 CAR-T cells (2×10^5^) pretreated with Dec (0.05, 0.1 μM) and Aza (0.5, 1 μM) for 6 days were co-cultured with 2×10^5^ MV4-11 cells in 100ul RPMI-1640 complete medium for 24 h. BD cytometric bead array (CBA) (BD Biosciences, Cat# 551809) was used to measure cytokines in supernatants.

### Repetitive stimulus analysis

MV4-11 cells treated with 10 μg/ml Mitomycin-C (Selleck, S8146) for 2 h were used to stimulate CD44v6 CAR-T cells pretreated with Dec 0.1 μM or Aza 1 μM. The E: T ratio and stimulation time were the same in each round. A round of stimulation was followed by staining CAR-T cells with trypan-blue and counting them under the microscope. CAR-T cells were isolated from MV4-11 cells that had been lysed. All CAR-T cells obtained in the last round were used as the initial cells for the next round of stimulation. The proliferation fold change is the ratio of cell counts after different rounds of stimulation compared to cell counts before stimulation.

### Animal experiments

Vital River Laboratory Animal Technology (Beijing, China) provided us with male five-week-old BALB/c-nu mice. Nanjing Biomedical Research Institute of Nanjing University (Nanjing, China) provided us with male five-week-old NOD-Prkdc^em26Cd52^il2rg^em26Cd22^/Nju (NCG) mice. These mice were housed in the individually ventilated cage (IVC) of the specific pathogen-free (SPF) environment at the Experimental Animal Center of Huazhong University of Science and Technology. All animal experiments were approved by the Institutional Animal Care and Use Committee of Huazhong University of Science and Technology.

Subcutaneous xenografted of 5×10^6^ luciferase-expressing MV4-11 (Luc^+^ MV4-11) cells were constructed in BALB/c-nu mice. These mice were randomly divided into 6 groups. Mice in the NT, CAR-T and Dec-pretreated-CD44v6 CAR-T (dCAR-T) groups were all intraperitoneally injected with 100 μl phosphate-buffered saline (PBS) on days 7, 8, 9, 14, 15, and 16, then intratumorally treated with NT, CAR-T or dCAR-T cells (5×10^6^) on days 10 and 17, respectively. Mice in the Dec, Dec+CAR-T and Dec+dCAR-T groups were all intraperitoneally injected with Dec 1 mg/kg on days 7, 8, 9, 14, 15, and 16, then intratumorally injected with 200 μl PBS, 5×10^6^ CAR-T or dCAR-T cells on days 10 and 17, respectively. An assessment of tumor burdens in mice was performed on days 7, 14, 21, and 28 by bioluminescence imaging (BLI). After the fourth BLI evaluation, the mice were humanely euthanized.

In NCG mice, MV4-11 cells (1×10^7^) and CD44v6 CAR-T cells (1×10^7^) pretreated with or without Dec or Aza were injected intravenically into NCG mice on day 0. After 3 weeks, peripheral blood of NCG mice was collected, and the percentage of CD44v6 CAR-T cells in peripheral blood was detected by flow cytometry.

### RNA-seq analysis

CD44v6 CAR-T cells from three healthy donors were treated with Dec or Aza for 6 days for RNA-seq analysis. Total RNA was isolated using the standard Trizol protocol. The RNA quality was determined with the Nano Drop and Agilent 2100 Bioanalyzer (Thermo Fisher Scientific, USA).The libraries for RNA-seq were sequenced on the DNBSEQ platform (BGI Genomics, Shenzhen, China). After RNA-seq, raw reads containing sequencing adapters with more than 5% unknown base or more than 20% low-quality base were removed using SOAPnuke software to obtain clean reads. Clean reads were aligned to the Homo-sapines reference genome (GRCh38.p13) sequence, taking the comparison ratio and the distribution of the reference sequence as conditions for further analysis. Quantitative analysis of gene expression levels in different samples was performed using RSEM software. Briefly, clean reads obtained from RNA-seq were mapped onto reference full-length transcripts using the Bowtie2 software. Subsequently, the expression level of each sample was calculated using RSEM software, and the read counts were normalized using fragment per kilobase of transcript per million fragments mapped (FPKM). In order to improve the accuracy of differentially expression genes (DEGs), we define genes with fold change >2 and Q-value ≤ 0.001, which are screened as significant DEGs. The R package pheatmap was used to perform hierarchical clustering analysis on the union set differential genes. DEGs were mapped to GO and KEGG databases to obtain annotated information by Phyper based on a hypergeometric test for further enrichment and classification analyses. The P values were corrected to Q values with a threshold Q value of ≤ 0.05 using a Bonferroni correction. And the RNA-seq data have been made publicly available in SRA under the accession number SRP417784.

### Statistical analysis

Results were expressed as mean ± SD and analyzed using GraphPad Prism 7. Student’s t test was used to compare two groups, and one-way or two-way ANOVA with Tukey’s multiple comparison test was used for comparing multiple groups. The *p* values <0.05 were considered statistically significant, and “ns” indicated no significance (*p* > 0.05).

## Results

### Decitabine and azacitidine increase the transduction efficiency and persistence of CD44v6 CAR-T

**Figure 1:**
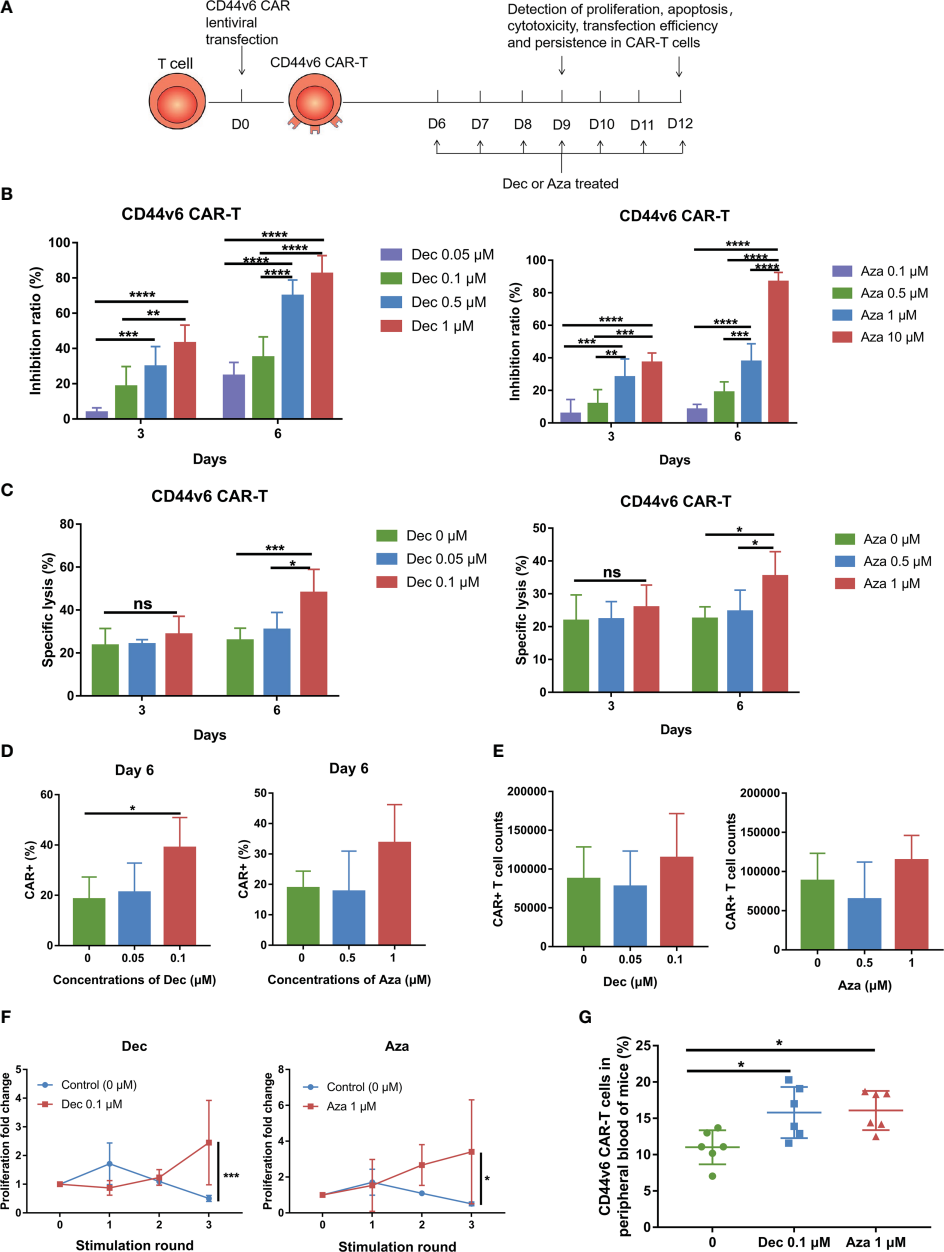
Dec and Aza treated CD44v6 CAR-T cells exhibit increased CAR+ cells and persistence. **(A)** Experimental design of CD44v6 CAR-T cells treated with Dec and Aza. **(B)** Inhibition ratio of CD44v6 CAR-T cells treated with various concentrations of Dec (left, n=6) and Aza (right, n=8) for 3 or 6 days. **(C)** Cytotoxicity of CD44v6 CAR-T cells treated with Dec (0.05, 0.1 μM) (left, n=6) and Aza (0.5, 1 μM) (right, n=5) for 3 or 6 days against MV4-11 cells in an E:T ratio of 10:1 for 24 h. **(D, E)** Percentage **(D)** and absolute number **(E)** of CAR+ cells in CD44v6 CAR-T cells treated with Dec (0.05, 0.1 μM) (n=4) and Aza (0.5, 1 μM) (n=5) for 6 days. **(F)** Proliferation fold of CD44v6 CAR-T cells treated with Dec 0.1uM and Aza 1uM for 6 days in response to repeated stimulation of MV4-11 cells (n=4). **(G)** CD44v6 CAR-T cells treated with or without Dec or Aza were injected intravenously into NCG mice simultaneously with MV4-11, and the percentage of CD44v6 CAR-T cells in peripheral blood was detected by flow cytometry 3 weeks later (n=6). CD44v6, CD44 isoform variant 6; CAR-T, chimeric antigen receptor T; Dec, decitabine; Aza, azacitidine. Data are depicted as the mean± SD. *p<.05; **p<.01; ***p<.001; ****p<.0001; ns, not significant.

Six days after transduction, Dec (0.05-1 μM) or Aza (0.1-10 μM) was added to CD44v6 CAR-T cells for 3 or 6 days. Dec and Aza were freshly added once a day at different final concentrations. Untreated CD44v6 CAR-T cells served as controls. Subsequently, we examined the proliferation, cytotoxicity, persistence, transduction efficiency, and apoptosis of CD44v6 CAR-T cells (**Figure 1A**). CAR-T cells proliferation was inhibited dose- and time- dependently by Dec or Aza (**Figure 1B**). To assess cytotoxicity, CD44v6 CAR-T cells received Dec (0.05, 0.1 μM) or Aza (0.5, 1 μM) treatment for 3 or 6 days before being co-cultured with MV4-11 cells. Cytotoxicity assay results showed that Dec 0.1 μM or Aza 1 μM treatment for 6 days significantly increased the cytotoxicity of CD44v6 CAR-T, which were not observed at treatment for 3 days (**Figure 1C**). Although we found that Dec and Aza slightly promoted CD44v6 CAR-T cell apoptosis (**Figure S1A**), Dec 0.1 μM and Aza 1 μM improved the transfection efficiency of CD44v6 CAR-T (**Figure 1D**) and increased the absolute number of CAR+ cells (**Figure 1E**). Therefore, we chose the drug concentration of Dec (0.05, 0.1 μM) and Aza (0.5, 1 μM), and the treatment time of 6 days for the subsequent experiments. In addition, repeated antigen stimulation experiments showed that CD44v6 CAR-T cells pretreated with Dec 0.1 μM or Aza 1 μM for 6 days showed accelerated expansion and enhanced persistence after three rounds of tumor cells stimulation (**Figure 1F**). The result that Dec or Aza enhanced the persistence of CD44v6 CAR-T cells was also confirmed *in vivo*. On day 0, MV4-11 cells (1×10^7^) and CD44v6 CAR-T cells (1×10^7^) pretreated with or without Dec or Aza were injected intravenically into NCG mice. After 3 weeks, peripheral blood of mice was collected to detect the percentage of CD44v6 CAR-T cells in peripheral blood. We found a higher percentage of Dec or Aza pretreated CD44v6 CAR-T than untreated CD44v6 CAR-T cells in mice (**Figure 1G**). These results suggested that Dec and Aza enhanced the function of CD44v6 CAR-T by increasing their transduction efficiency, absolute number of CAR+ cells and persistence. And CD44v6 CAR-T cells were more sensitive to Dec.

### Decitabine and azacitidine promote activation and memory phenotype of CD44v6 CAR-T

**Figure 2:**
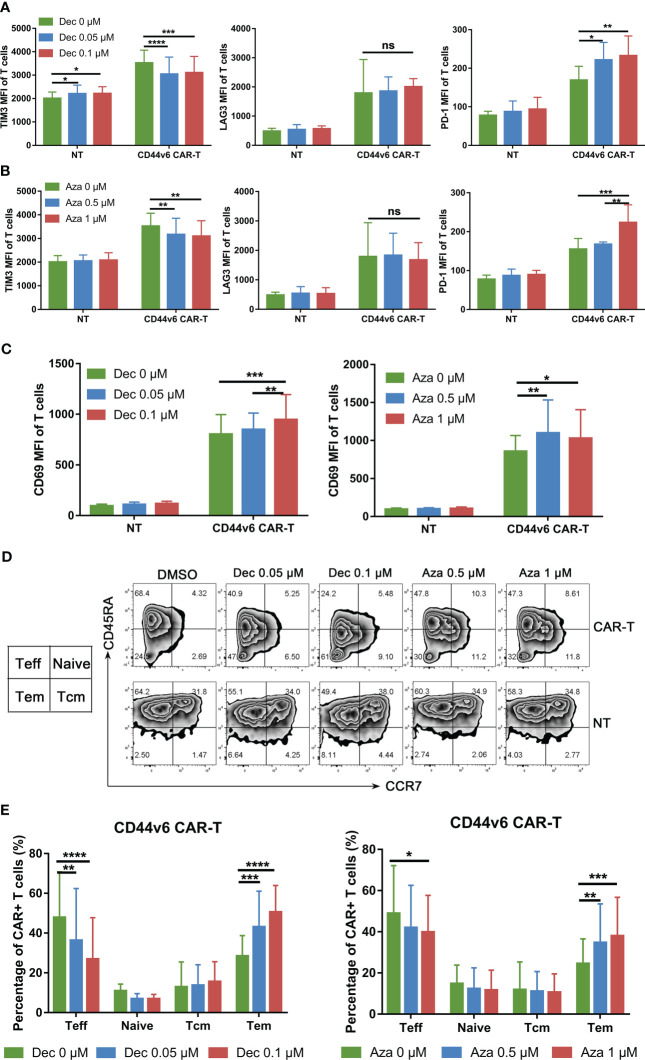
Effects of Dec and Aza on the exhaustion and phenotypic differentiation of CD44v6 CAR-T. **(A, B)** TIM3, LAG3 and PD-1 expression levels of non-transduced T (NT) and CD44v6 CAR-T cells treated with Dec (0.05, 0.1 μM) **(A)** and Aza (0.5, 1 μM) **(B)** for 6 days (n=4). **(C)** CD69 expression level of NT and CD44v6 CAR-T cells treated with Dec (0.05, 0.1 μM) (left) and Aza (0.5, 1 μM) (right) for 6 days (n=4) **(D, E)** CCR7 and CD45RA surface staining was performed on NT and CD44v6 CAR-T cells (n=5) treated with Dec (0.05, 0.1 μM) and Aza (0.5, 1 μM) for 6 days, representative flow cytometry is shown in D, the changes of phenotype of CD44v6 CAR-T cells are indicated in **(E)** MFI, mean fluorescence intensity; Teff, effector T cells; Tcm, central memory T cells; Tem, effector memory T cells. Data are depicted as the mean ± SD. *p<.05; **p<.01; ***p<.001; ****p<.0001; ns, not significant.

It has been demonstrated that DNMT3A-mediated *de novo* DNA methylation program induces T cell exhaustion and negatively influences T cell differentiation and function (22–24). Our results showed that low-dose Dec and Aza inhibited DNMT3A mRNA expression in CD44v6 CAR-T cells (**Figure S1B**). Dec and Aza also inhibited the proliferation of NT cells which served as controls (**Figure S1C**). Flow cytometry results showed that Dec and Aza inhibited TIM3 expression and promoted LAG3 expression, but had no effect on PD-1 expression in CD44v6 CAR-T cells (**Figures 2A, B**).Although Dec or Aza had inconsistent effects on the exhaustion markers of CD44v6 CAR-T cells, Dec and Aza promoted the expression of CD69, an activation marker of CD44v6 CAR-T cells (**Figure 2C**). Dec- or Aza-treated NT or CD44v6 CAR-T cells had fewer effector T cells (Teff) and more effector memory T cells (Tem) compared to untreated NT and CD44v6 CAR-T cells (**Figures 2D, E**, **S1D**). These data indicated that Dec and Aza promoted activation and Tem differentiation of CD44v6 CAR-T.

### Decitabine and azacitidine enhance the cytotoxicity of CD44v6 CAR-T

**Figure 3:**
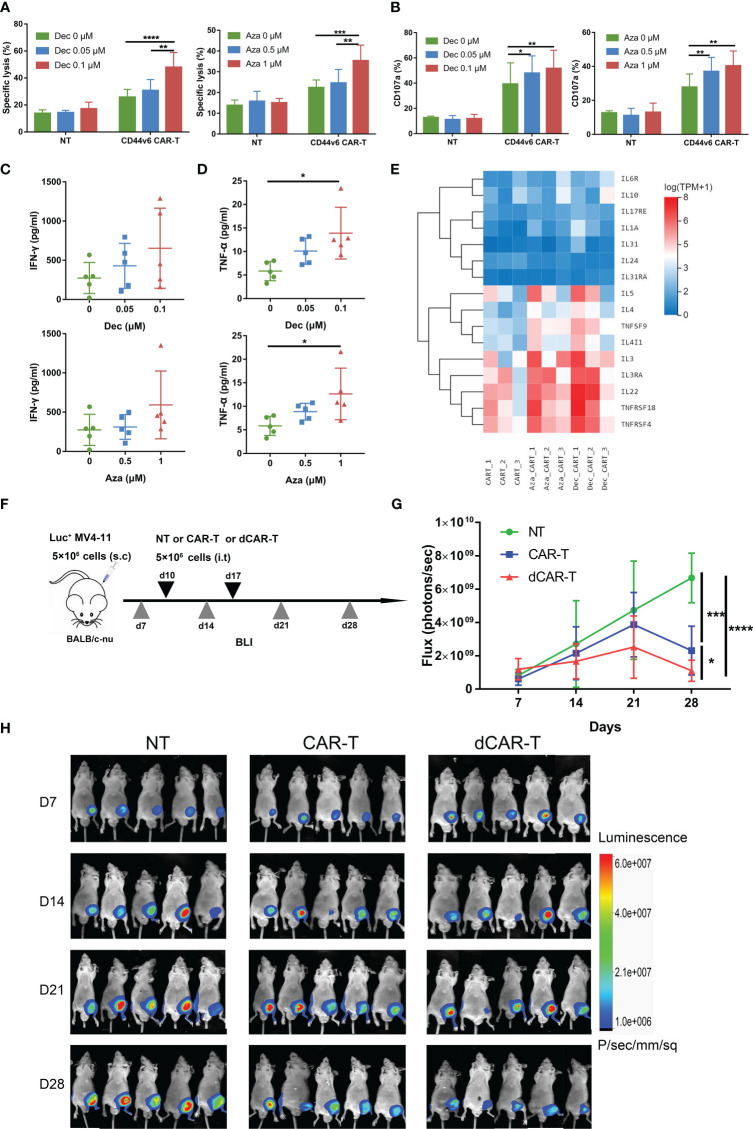
Dec and Aza pretreated CD44v6 CAR-T cells exhibit enhanced anti-tumor ability *in vitro* and *in vivo*. **(A)** Cytotoxicity of NT and CD44v6 CAR-T cells treated with Dec (0.05, 0.1 μM) (left, n=6) and Aza (0.5, 1 μM) (right, n=5) for 6 days against MV4-11 cells in an E:T ratio of 10:1 for 24 h. **(B)** Percentage of CD107a^+^ NT and CD44v6 CAR-T cells treated with Dec (0.05, 0.1 μM) (left, n=3) and Aza (0.5, 1 μM) (right, n=4) for 6 days against MV4-11 cells in an E:T ratio of 1:1 for 16 h. **(C, D)** Cytokine IFN-γ **(C)** and TNF-α **(D)** release of CD44v6 CAR-T cells (n=5) treated with Dec (0.05, 0.1 μM) and Aza (0.5, 1 μM) for 6 days against MV4-11 cells in an E:T ratio of 1:1 for 24 h. **(E)** Heatmap shows expression of elevated tumor necrosis factor and interleukin genes of CD44v6 CAR-T cells treated with Dec 0.1 μM and Aza 1 μM for 6 days in RNA-seq. **(F)** Schematic of Dec pretreated CD44v6 CAR-T treatment of MV4-11 cells xenografts mice. BALB/c-nu mice were subcutaneously injected with 5×10^6^ MV4-11-firefly luciferase (Luc^+^ MV4-11) cells on day 0. NT (n=5), CD44v6 CAR-T (n=5) and Dec pretreated CD44v6 CAR-T (dCAR-T, n=5) (5×10^6^) were intratumorally injected on day 10 and day 17. Tumor burden was analyzed by BLI on days 7, 14, 21 and 28. **(G, H)** Graph **(G)** and BLI images **(H)** showing the progress of tumor burden at the indicated time point. Luc^+^ MV4-11, luciferase-expressing MV4-11 cells; dCAR-T, Dec pretreated CD44v6 CAR-T; BLI, bioluminescence imaging. Data are depicted as the mean ± SD. *p<.05; **p<.01; ***p<.001; ****p<.0001; ns, not significant.

To assess cytotoxicity *in vitro*, CD44v6 CAR-T or NT cells received Dec (0.05, 0.1μM) or Aza (0.5, 1μM) treatment for 6 days before being co-cultured with MV4-11 cells. After 24 h, we found that Dec 0.1 μM and Aza 1 μM improved the cytotoxicity of CD44v6 CAR-T, but not in NT cells (**Figures 3A**, **S2A**). Compared to untreated CD44v6 CAR-T, Dec or Aza pretreated CD44v6 CAR-T cells expressed more CD107a (**Figure 3B**) and released more cytokines (**Figures 3C, D**, **S2B, C**). RNA-seq data also demonstrated that Dec and Aza upregulated the expression of multiple tumor necrosis factor and interleukin genes in CD44v6 CAR-T cells (**Figure 3E**). We then evaluated the anti-tumor capacity of Dec-pretreated-CD44v6 CAR-T (dCAR-T) within the body, based on the promotion of anti-tumor activity by Dec *in vitro*. Subcutaneous xenografted of 5×10^6^ Luc^+^ MV4-11 cells were constructed in BALB/c-nu mice, that were subsequently treated with 5×10^6^ NT, CD44v6 CAR-T and dCAR-T cells on days 10 and 17. The tumor burden was assessed weekly using BLI (**Figure 3F**). BLI results showed that dCAR-T cells had the strongest effect in preventing tumor progression in all three groups (**Figures 3G, H**). According to these data, Dec and Aza promoted the anti-tumor activity of CD44v6 CAR-T cells both *in vitro* and *in vivo*.

### Decitabine and azacitidine affect the transcriptome of CD44vv6 CAR-T cells

**Figure 4:**
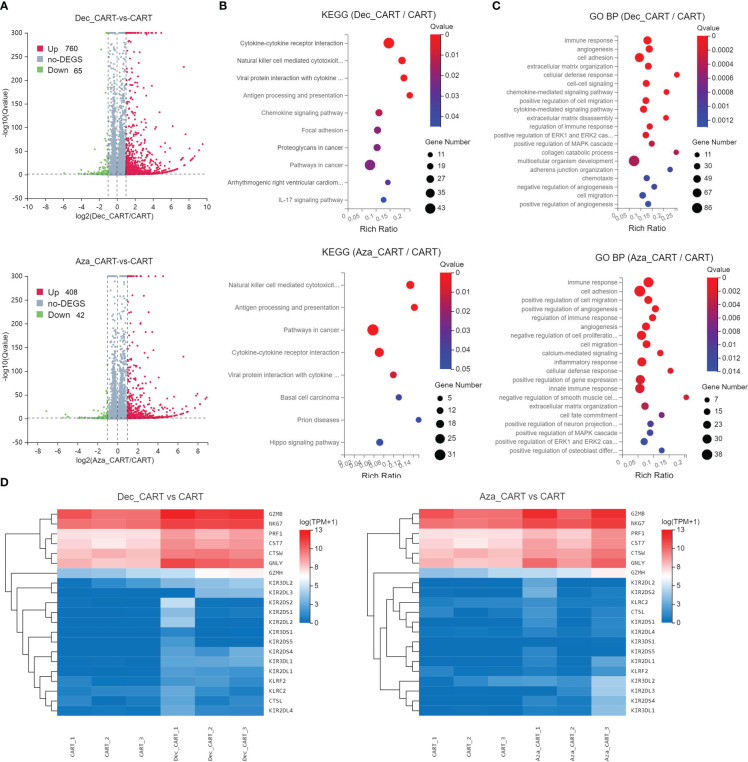
Analysis of differentially expressed genes and functions of CD44v6 CAR-T cells treated with Dec and Aza. CD44v6 CAR-T cells (n=3) treated with Dec 0.1 μM (Dec_CAR-T) and Aza 1 μM (Aza_CAR-T) for 6 days were measured by RNA-seq. Untreated CD44v6 CAR-T cells were used as controls (CAR-T). **(A)** Volcano plot of differentially expressed genes (DEGs) in Dec_CAR-T (above) or Aza_CAR-T (below) compared with CAR-T. Red dots represent genes upregulated in Dec_CAR-T or Aza_CAR-T (Qvalue < 0.05 and log2(fold change) ≥1), while blue dots represent genes downregulated in Dec_CAR-T or Aza_CAR-T (Qvalue < 0.05 and log2(fold change) ≤ −1). **(B, C)** Significantly enriched (Qvalue < 0.05) KEGG pathways **(B)** and GO BP **(C)** of all DEGs from Dec_CAR-T (above) or Aza_CAR-T (below) compared to CAR-T. Dot color indicates the statistical significance of enrichment (Qvalue), and dot size represents gene count enriched in each term. **(D)** Heatmap of differentially expressed cytotoxicity-related genes (Qvalue < 0.001 and log2(fold change) ≥1) from Dec_CAR-T (left) or Aza_CAR-T (right) compared to CAR-T. KEGG, Kyoto Encyclopedia of Genes and Genomes; GO, Gene Ontology; BP, biological process.

We performed RNA-seq to explore the transcriptome differences between Dec 0.1μM or Aza 1 μM treated CD44v6 CAR-T and untreated CD44v6 CAR-T. We found 825 and 450 differentially expressed genes (DEGs) in these two comparisons (Dec_CART vs. CART, and Aza_CART vs. CART), respectively (**Figure 4A**). The DEGs were significantly enriched in the Natural killer cell mediated cytotoxicity, Antigen processing and presentation, Cytokine-cytokine receptor interaction, and Chemokine signaling pathway, etc (**Figure 4B**). Further, GO BP enrichment results also showed that these DEGs involved immune response, cell adhesion, cell-cell signaling, and cell migration, etc (**Figure 4C**). Cytotoxicity-related genes such as *GZMB*, *NKG7*, *PRF1*, *CST7*, *CTSW*, *GNLY*, *GZMH*, *KIR3DL2*, *KIR2DL3*, and *KIR2DS4* were significantly upregulated in Dec- or Aza-treated CD44v6 CAR-T cells (**Figure 4D**). These RNA-seq results again demonstrated that Dec and Aza strengthened the function of CD44v6 CAR-T cells.

### Decitabine and azacitidine promote CD44v6 expression in AML cells

**Figure 5:**
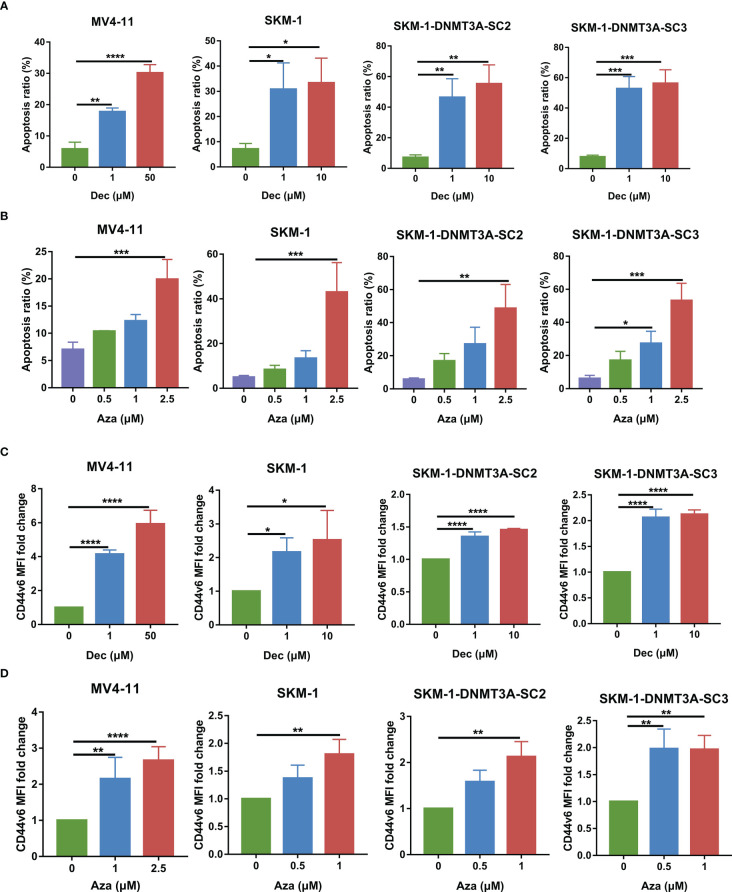
Dec and Aza treatment promotes the apoptosis and CD44v6 expression on AML cells. **(A, B)** Apoptosis of MV4-11, SKM-1, SKM-1-DNMT3A-SC2 and SKM-1-DNMT3A-SC3 cells (n=3) after treatment with different concentrations of Dec **(A)** and Aza **(B)** for 3 days. **(C, D)** CD44v6 MFI fold change of MV4-11, SKM-1, SKM-1-DNMT3A-SC2 and SKM-1-DNMT3A-SC3 cells (n=4) after treatment with different concentrations of Dec **(C)** and Aza **(D)** for 3 days. CD44v6 MFI fold change is the ratio of CD44v6 MFI of Dec and Aza treated CD44v6 CAR-T divided by the CD44v6 MFI of untreated CD44v6 CAR-T. SKM-1-DNMT3A-SC2 and SKM-1-DNMT3A-SC3: two clones of DNMT3A-R882H mutant of SKM-1 cells. MFI, mean fluorescence intensity. Data are depicted as the mean ± SD. *p<.05; **p<.01; ***p<.001; ****p<.0001; ns, not significant.

Studies have shown that Dec and Aza can enhance immunotherapy by upregulating the expression of tumor-associated antigens on AML and other cancer cells [14-18]. We then investigated the effect of Dec and Aza on AML tumor cells. Dec and Aza were freshly added once a day at different final concentrations for 3 days. In our previous study, SKM-1-DNMT3A-SC2 and SKM-1-DNMT3A-SC3 are clones of DNMT3A-R882H mutant cells generated by lentiviral transduction of SKM-1 cells (9). Dec and Aza inhibited proliferation and promoted apoptosis of AML cell lines MV4-11, SKM-1, and DNMT3A mutant SKM-1 cells dose-dependently (**Figures 5A, B**, **S3A, B**). Dec specifically promoted the apoptosis of DNMT3A mutant SKM-1 cells. Our previous study revealed that SKM-1 cells expressed low levels of CD44v6, DNMT3A mutation promoted CD44v6 expression in DNMT3A mutant SKM-1 cells, and FLT3 mutant MV4-11 cells expressed high levels of CD44v6 (9). No matter whether FLT3 or DNMT3A mutations were present, Dec and Aza promoted CD44v6 expression in these AML cells (**Figures 5C, D**, **S3C**).

### Decitabine and azacitidine pretreated AML cells improve the anti-tumor ability of CD44v6 CAR-T

**Figure 6:**
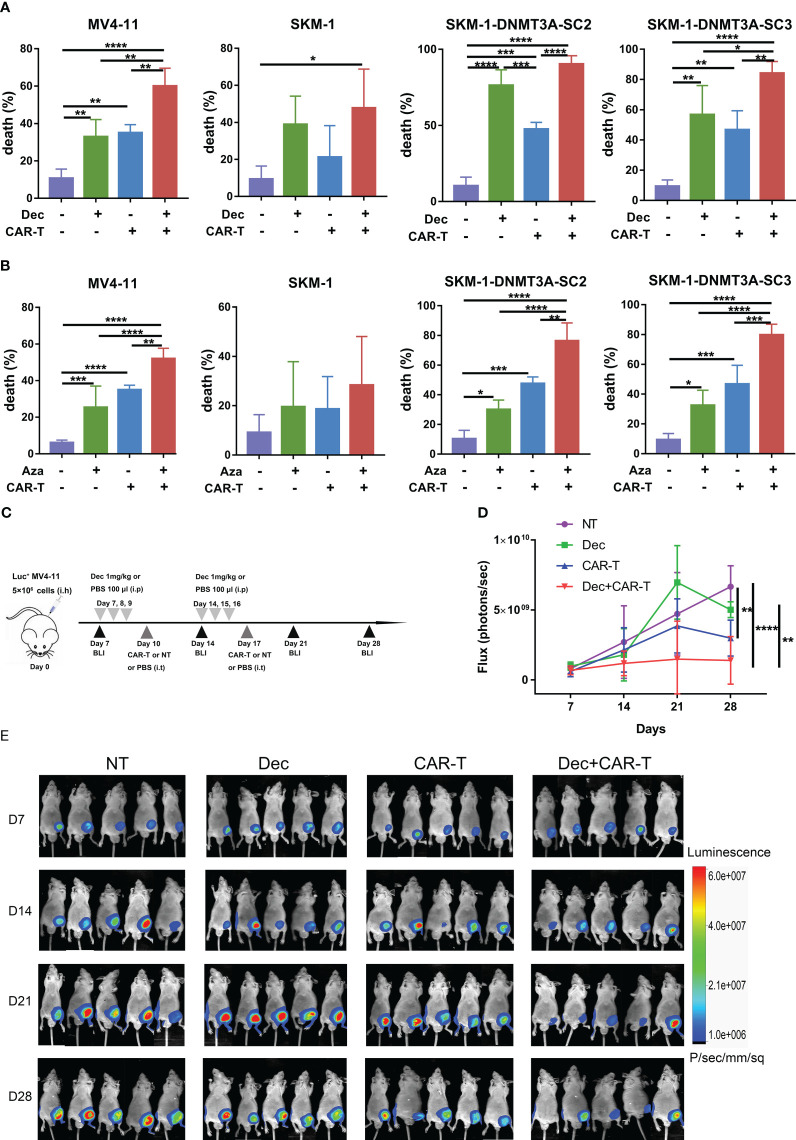
Effect of Dec and Aza pretreated AML cells on the anti-tumor capacity of CD44v6 CAR-T *in vitro* and *in vivo*. **(A, B)** Dec **(A)** and Aza **(B)** pretreated AML cells for 3 days were then co-cultured with CD44v6 CAR-T at an E:T of 10:1 for 24 **(h)** Flow cytometric detected the percentage of dead cells in MV4-11, SKM-1, SKM-1-DNMT3A-SC2 and SKM-1-DNMT3A-SC3 cells (n≥4) pretreated with Dec or Aza alone, CD44v6 CAR-T alone, and Dec or Aza in combination with CD44v6 CAR-T. **(C)** Dec pretreated MV4-11 cell xenograft model. BALB/c-nu mice were subcutaneously injected with 5×10^6^ MV4-11-firefly luciferase (Luc^+^ MV4-11) cells on day 0. Dec 1 mg/kg or 100 μl PBS were injected intraperitoneally on days 7, 8, 9, 14, 15 and 16. CD44v6 CAR-T cells (5 × 10^6^), NT cells (5 × 10^6^), or 200 μl PBS were intratumorally injected on day 10 and day 17. Tumor burden was analyzed by BLI on days7, 14, 21 and 28. **(D, E)** Graph **(D)** and BLI images **(E)** showing the progress of tumor burden at the indicated time point. Data are depicted as the mean ± SD. *p<.05; **p<.01; ***p<.001; ****p<.0001; ns, not significant.

To investigate whether Dec or Aza pretreatment of AML cells could enhance the anti-tumor ability of CD44v6 CAR-T, AML cells were treated with Dec 1 μM or Aza 1 μM for 3 days and then incubated with CD44v6 CAR-T cells at an E: T ratio of 10:1 for 24 h. Cytotoxicity assays showed that Dec or Aza pretreated AML combined with CD44v6 CAR-T had the highest cytotoxicity compared to Dec or Aza alone or CD44v6 CAR-T alone (**Figures 6A, B**). This finding was also observed *in vivo*. Subcutaneous inoculation of Luc^+^ MV4-11 cells was performed on BALB/c-nu mice and the tumor burden was assayed by BLI. The protocols using these agents were shown in **Figure 6C**. BLI results showed that Dec followed by CD44v6 CAR-T therapy had the strongest ability to prevent tumor progression than Dec or CAR-T therapy alone (**Figures 6D, E**). These data proved that Dec or Aza pretreated AML enhanced the anti-tumor ability of CD44v6 CAR-T.

### Combination of decitabine and azacitidine pretreated AML cells and pretreated CD44v6 CAR-T enhanced the anti-tumor capacity

**Figure 7:**
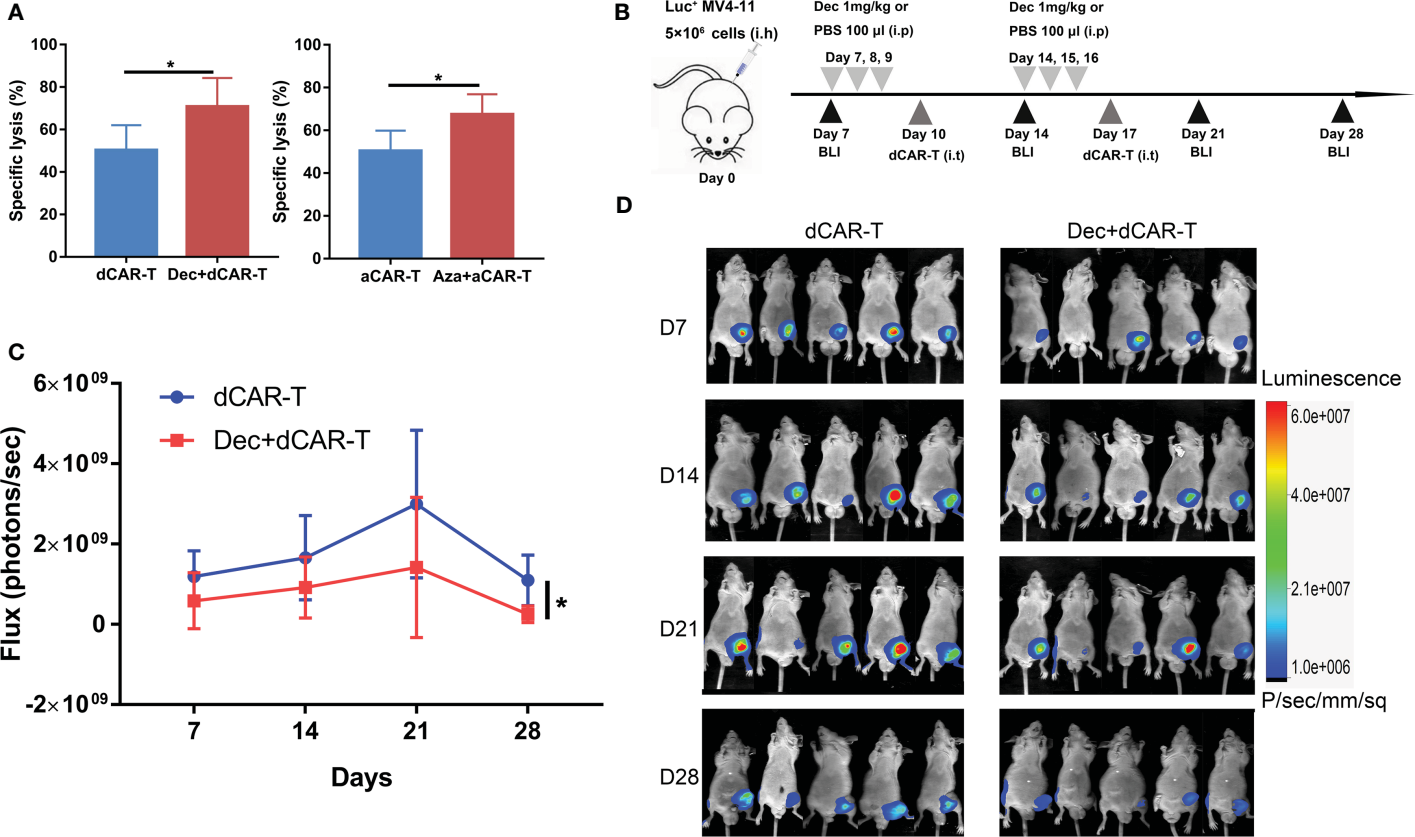
The anti-tumor ability of Dec or Aza pretreated CD44v6 CAR-T against Dec and Aza pretreated AML *in vitro* and *in vivo*. **(A)** Cytotoxicity of Dec (left) and Aza (right) pretreated CD44v6 CAR-T (dCAR-T, aCAR-T, n=6) to MV4-11 cells pretreated with or without Dec and Aza in an E:T ratio of 10:1 for 24 h. **(B)** Schematic of Dec pretreated CD44v6 CAR-T treatment of Dec pretreated MV4-11 cells xenografts mice. BALB/c-nu mice were subcutaneously injected with 5×10^6^ MV4-11-firefly luciferase (Luc^+^ MV4-11) cells on day 0. Dec 1 mg/kg or 100 μl PBS were intraperitoneally injected on day 7, 8, 9, 14, 15 and 16. Then dCAR-T cells (5×10^6^) were intratumorally injected on day 10 and day 17. Tumor burden was analyzed by BLI on days 7, 14, 21 and 28. **(C, D)** Graph **(C)** and BLI images **(D)** showing the progress of tumor burden at the indicated time point. dCAR-T: Dec pretreated CD44v6 CAR-T; aCAR-T: Aza pretreated CD44v6 CAR-T. Data are depicted as the mean± SD. *p<.05; ns, not significant.

The combination of Dec or Aza pretreated AML and pretreated CD44v6 CAR-T (dCAR-T, aCAR-T) significantly enhanced the anti-tumor capacity compared to dCAR-T or aCAR-T alone *in vitro* (**Figure 7A**), which was also verified *in vivo*. Subcutaneous BALB/c-nu mouse models inoculated with Luc^+^ MV4-11 cells were intraperitoneally injected with 1 mg/kg Dec or 100 ul PBS on days 7, 8, 9, 14, 15, and 16, followed by intratumoral injection of dCAR-T on days 10 and 17 (**Figure 7B**). We found that Dec+dCAR-T therapy significantly eliminated tumor burden in mice compared to dCAR-T therapy (**Figures 7C, D**). These results demonstrated that the combination of Dec or Aza pretreated AML and pretreated CAR-T had a higher anti-tumor capacity compared to pretreated CAR-T alone.

### Security of Dec and Aza pretreatment

**Figure 8:**
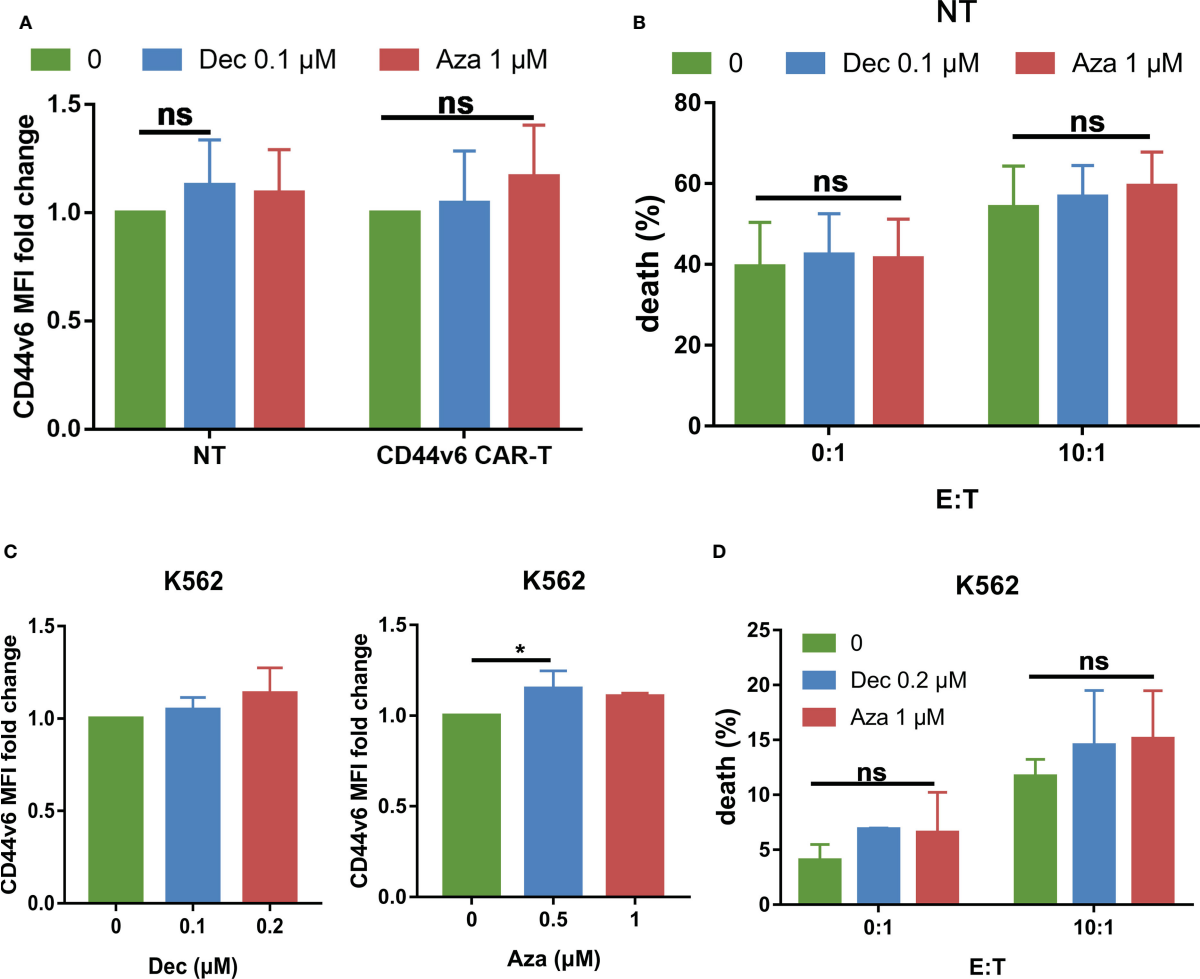
Effects of Dec and Aza on CD44v6 expression and safety in T cells and K562 cells. **(A)** CD44v6 MFI fold change of NT and CD44v6 CAR-T cells (n=4) after treatment with Dec and Aza for 6 days. **(B)** Cytotoxicity of CD44v6 CAR-T cells (n=8) against NT cells pretreated with Dec 0.1 μM or Aza 1 μM for 6 days in E:T ratios of 0: 1 and 10:1 for 24 h. **(C)** CD44v6 MFI fold change of K562 cells (n=4) after treatment with Dec and Aza for 3 days. **(D)** Cytotoxicity of CD44v6 CAR-T cells against K562 cells pretreated with Dec 0.2 μM or Aza 1 μM for 3 days in E:T ratios of 0: 1 and 10:1 for 24 h (n=3). Data are depicted as the mean± SD. *p<.05; ns, not significant.

Our previous study showed that activated T cells express low levels of CD44v6, so there is transient fratricide of CD44v6 CAR-T cells. Does Dec or Aza pretreatment of NT or CD44v6 CAR-T cells increase fratricide? We found that Dec or Aza treatment of NT or CD44v6 CAR-T cells for 6 days slightly increased the expression of CD44v6 in NT and CD44v6 CAR-T cells (**Figure 8A**). Next, NT cells pretreated with or without Dec, Aza were co-cultured with CD44v6 CAR-T cells at E: T ratios of 0:1 and 10:1 for 24 h. We found that Dec and Aza pretreatment of NT cells did not significantly increase the cytotoxicity of CD44v6 CAR-T cells against NT cells (**Figure 8B**). K562 cells are chronic myeloid leukemia cells that do not express CD44v6. Treatment of K562 cells with Dec and Aza for 3 days inhibited their proliferation (**Figure S3D**) and slightly increased their CD44v6 expression (**Figure 8C**), but did not significantly increase the cytotoxicity of CD44v6 CAR-T cells against K562 cells (**Figure 8D**). These data suggested that Dec and Aza pretreatment of T cells or other cells that do not express CD44v6 did not increase fratricide and off-target toxicity of CD44v6 CAR-T cells.

## Discussion

Our previous study demonstrated that CD44v6 was an optimal target for AML, and CD44v6 CAR-T showed specific and effective anti-leukemic ability against AML (9). However, the early expansion of CD44v6 CAR-T cells was limited by the transient fratricide. Expansion of CD44v6 CAR-T by extending culture time resulted in differentiation of effector T cells. Study shows effector T cells are detrimental to the anti-tumor capacity and persistence of CAR-T (27). Therefore, new strategies are needed to improve the functions of CD44v6 CAR-T. Here, we found Dec and Aza strengthened the cytotoxicity of CD44v6 CAR-T by increasing their persistence and the number of CAR+ cells, promoting their activation, and facilitating their memory phenotype. Dec and Aza also enhanced the sensitivity of AML cells to CD44v6 CART-mediated cytotoxicity by upregulating the expression of CD44v6 in AML cells. And Dec and Aza pretreatment did not increase fratricide and off-target toxicity of CD44v6 CAR-T cells.

Dec or Aza are demonstrated to promote the anti-tumor ability of CD19 CAR-T and CD123 CAR-T (28, 29). Does Dec or Aza promote the anti-tumor ability of CD44v6 CAR-T? Low doses of Dec and Aza inhibit DNA methylation, while high doses induce apoptosis (20, 24). To avoid inhibiting CAR-T cell activity, we used low doses of Dec (0.05-1 μM) and Aza (0.1-10 μM) to investigate their effects on CD44v6 CAR-T proliferation. We found that Dec and Aza inhibited CD44v6 CAR-T proliferation in a dose-dependent and time-dependent manner. CD44v6 CAR-T cells were more sensitive to Dec. Stübig et al. also revealed that Aza inhibited T cell proliferation dose-dependently (30). Although Dec and Aza inhibited CD44v6 CAR-T proliferation and slightly promoted apoptosis, Dec 0.1 μM and Aza 1 μM treatment of CD44v6 CAR-T for 6 days significantly improved their transduction efficiency, absolute number of CAR+ cells, and anti-tumor capacity *in vitro* and *in vivo*. Moreover, CD44v6 CAR-T cells pretreated with Dec or Aza showed enhanced persistence *in vitro* and *in vivo*. These data suggested that Dec or Aza pretreatment of CD44v6 CAR-T significantly enhanced its anti-tumor ability against AML.

Several factors determine whether CAR-T cells are effective. HMAs can reverse the exhaustion associated with the *de novo* DNA methylation (22). However, in our study, Dec and Aza inhibited TIM3 expression and promoted LAG3 expression in CD44v6 CAR-T cells. The effects of Dec and Aza on the exhaustion markers of CD44v6 CAR-T cells were not consistent. Since the expression level of TIM3 was significantly higher than that of LAG3, we speculated that the effect of Dec and Aza on the exhaustion of CD44v6 CAR-T cells was dominated by a decrease in TIM3, which needs to be verified by follow-up experiments in the future. Moreover, Dec and Aza promoted CD44v6 CAR-T cells activation, which helped to improve the function of CD44v6 CAR-T cells. Tumors are eliminated more effectively by naive or memory T cells than effector T cells (27). According to clinical research, CD19 CAR-T cells from patients who responded well to CAR-T for CLL had more memory T cells, while CD19 CAR-T cells from non-responders had more effector T cells (31). Knockout of the *DNMT3A* gene in T cells drives T cells to a higher proportion of memory phenotypes (32), and the DNMT3A inhibitors Dec and Aza have similar effects on T cells (28, 29). Our study also found that CD44v6 CAR-T cells treated with Dec or Aza had more memory T cells and fewer effector T cells than untreated CAR-T cells. Therefore, we speculated that Dec and Aza promoted the anti-tumor capacity of CD44v6 CAR-T cells by promoting activation and memory phenotype differentiation of CD44v6 CAR-T cells.

Dec and Aza have achieved significant efficacy in the induction of differentiation therapy in AML (19–21, 33–35). Low doses of Dec or Aza produced the best clinical response in hematologic malignancies, while high doses inhibited clinical response. Low doses of Dec and Aza induce differentiation, reduce proliferation and increase apoptosis by altering the gene expression profile of tumor cells (20, 34). In MV4-11, SKM-1 and DNMT3A mutant SKM-1 cells, low doses of Dec and Aza inhibited proliferation and induced apoptosis. Furthermore, Dec especially promoted apoptosis in DNMT3A mutant SKM-1 cells, which may be related to their high expression of DNMT3A mRNA (9, 36). Several studies have demonstrated that Dec and Aza promote the efficacy of immunotherapy by upregulating the expression of tumor-associated antigens in AML or other tumors. Our results are consistent with this, Dec and Aza enhanced the cytotoxicity of CD44v6 CAR-T against AML cells *in vitro* and *in vivo*, by promoting CD44v6 expression in AML cells regardless of FLT3 or DNMT3A mutations. Therefore, we concluded that Dec and Aza improved the anti-tumor ability of CD44v6 CAR-T cells by upregulating CD44v6 expression of AML cells.

Based on the two pretreatment protocols mentioned above, Dec or Aza pretreatment of CD44v6 CAR-T cells or AML cells both promoted the anti-tumor capacity of CD44v6 CAR-T cells. The combination of Dec or Aza pretreated CD44v6 CAR-T cells and pretreated AML was then investigated and found to have the strongest anti-tumor capacity.

In summary, this study presents the first evidence that the combination of Dec or Aza with CD44v6 CAR-T cells is potent for treating AML. Dec and Aza strengthened the anti-tumor capacity of CD44v6 CAR-T cells towards AML by increasing the counts of CAR+ cells and persistence, promoting activation and memory phenotype of CD44v6 CAR-T cells, and upregulating CD44v6 expression in AML cells. Thus, we proved that Dec or Aza combined with CD44v6 CAR-T is a promising therapy for AML.

## Data availability statement

The datasets presented in this study can be found in online repositories. The names of the repository/repositories and accession number(s) can be found below: NCBI *via* accession ID: PRJNA924523.

## Ethics statement

After obtaining written informed consent in accordance with the Declaration of Helsinki and approval by the Institutional Review Board of Tongji Medical College and the Hubei committee, peripheral blood of healthy donor was obtained from Union Hospital, Tongji Medical College, Huazhong University of Science and Technology. All animal experiments were approved by the Institutional Animal Care and Use Committee of Huazhong University of Science and Technology, and strictly followed the animal regulations of Hubei Province.

## Author contributions

FM, JW, and YY: Conceptualization, Funding acquisition, Writing - review and editing. LT and YK: Experimental implementation, Data curation, Formal analysis, Writing - original draft. HW and TS: Investigation, Methodology. JZ: Supervision. XZ and PZ: Project administration. NJ: Validation. HM and YL: Software. All authors contributed to the article and approved the submitted version.

## References

[1] Yang X Wang J. Precision therapy for acute myeloid leukemia. J Hematol Oncol (2018) 11(1):3. doi: 10.1186/s13045-017-0543-7 PMC5755341 29301553

[2] Thol F Ganser A. Treatment of relapsed acute myeloid leukemia. Curr Treat Option On (2020) 21(8):66. doi: 10.1007/s11864-020-00765-5 PMC7324428 32601974

[3] Daver N Schlenk RF Russell NH Levis MJ. Targeting FLT3 mutations in AML: review of current knowledge and evidence. Leukemia (2019) 33(2):299–312. doi: 10.1038/s41375-018-0357-9 30651634 PMC6365380

[4] DiNardo CD Tiong IS Quaglieri A MacRaild S Loghavi S Brown FC. Molecular patterns of response and treatment failure after frontline venetoclax combinations in older patients with AML. Blood (2020) 135(11):791–803. doi: 10.1182/blood.2019003988 31932844 PMC7068032

[5] Borot F Wang H Ma Y Jafarov T Raza A Ali AM. Gene-edited stem cells enable CD33-directed immune therapy for myeloid malignancies. Proc Natl Acad Sci (2019) 116(24):11978–87. doi: 10.1073/pnas.1819992116 PMC6575599 31138698

[6] Arcangeli S Rotiroti MC Bardelli M Simonelli L Magnani CF Biondi A. Balance of anti-CD123 chimeric antigen receptor binding affinity and density for the targeting of acute myeloid leukemia. Mol Ther (2017) 25(8):1933–45. doi: 10.1016/j.ymthe.2017.04.017 PMC5542631 28479045

[7] Wang J Chen S Xiao W Li W Wang L Yang S. CAR-T cells targeting CLL-1 as an approach to treat acute myeloid leukemia. J Hematol Oncol (2018) 11(1):7. doi: 10.1186/s13045-017-0553-5 PMC5761206 29316944

[8] Wang Y Xu Y Li S Liu J Xing Y Xing H. Targeting FLT3 in acute myeloid leukemia using ligand-based chimeric antigen receptor-engineered T cells. J Hematol Oncol (2018) 11(1):60. doi: 10.1186/s13045-018-0603-7 PMC5930553 29716633

[9] Tang L Huang H Tang Y Li Q Wang J Li D. CD44v6 chimeric antigen receptor T cell specificity towards AML with FLT3 or DNMT3A mutations. Clin Trans Med (2022) 12(9):e1043. doi: 10.1002/ctm2.1043 PMC9513046 36163632

[10] Monica Casucci BNDR Pietro Genovese BGFG Aurore Saudemont CBBS Chiara Bonini AAB. CD44v6-targeted T cells mediate potent antitumor effects against acute myeloid leukemia and multiple myeloma. Blood (2013) 122(20):3461–72. doi: 10.1182/blood-2013-04 24016461

[11] Heider K Kuthan H Stehle G Munzert G. CD44v6: a target for antibody-based cancer therapy. Cancer Immunology Immunotherapy (2004) 53(7):567–79. doi: 10.1007/s00262-003-0494-4 PMC11032850 14762695

[12] Shah NN Fry TJ. Mechanisms of resistance to CAR T cell therapy. Nat Rev Clin Oncol (2019) 16(6):372–85. doi: 10.1038/s41571-019-0184-6 PMC8214555 30837712

[13] Majzner RG Mackall CL. Clinical lessons learned from the first leg of the CAR T cell journey. Nat Med (2019) 25(9):1341–55. doi: 10.1038/s41591-019-0564-6 31501612

[14] Maus MBLH. Non-cleavable hinge enhances avidity and expansion of CAR-T cells for acute myeloid leukemia. Cancer Cell (2022) 40:494–508. doi: 10.1016/j.ccell.2022.04.001 35452603 PMC9107929

[15] Cao X Dai H Cui Q Li Z Shen W Pan J. CD7-directed CAR T-cell therapy: a potential immunotherapy strategy for relapsed/refractory acute myeloid leukemia. Exp Hematol Oncol (2022) 11(1):67. doi: 10.1186/s40164-022-00318-6 PMC9523980 36175988

[16] Grunewald CM Haist C König C Petzsch P Bister A Nößner E. Epigenetic priming of bladder cancer cells with decitabine increases cytotoxicity of human EGFR and CD44v6 CAR engineered T-cells. Front Immunol (2021) 12:782448. doi: 10.3389/fimmu.2021.782448 34868059 PMC8637820

[17] Wong KK Hassan R Yaacob NS. Hypomethylating agents and immunotherapy: therapeutic synergism in acute myeloid leukemia and myelodysplastic syndromes. Front Oncol (2021) 11:624742. doi: 10.3389/fonc.2021.624742 33718188 PMC7947882

[18] Klar AS Gopinadh J Kleber S Wadle A Renner C. Treatment with 5-Aza-2’-Deoxycytidine induces expression of NY-ESO-1 and facilitates cytotoxic T lymphocyte-mediated tumor cell killing. PloS One (2015) 10(10):e139221. doi: 10.1371/journal.pone.0139221 PMC4598131 26447882

[19] Stahl M Menghrajani K Derkach A Chan A Xiao W Glass J. Clinical and molecular predictors of response and survival following venetoclax therapy in relapsed/refractory AML. Blood Adv (2021) 5(5):1552–64. doi: 10.1182/bloodadvances.2020003734 PMC7948282 33687434

[20] Issa JJ Garcia-Manero G Giles FJ Mannari R Thomas D Faderl S. Phase 1 study of low-dose prolonged exposure schedules of the hypomethylating agent 5-aza-2′-deoxycytidine (decitabine) in hematopoietic malignancies. Blood (2004) 103(5):1635–40. doi: 10.1182/blood-2003-03-0687 14604977

[21] DiNardo CD Pratz K Pullarkat V Jonas BA Arellano M Becker PS. Venetoclax combined with decitabine or azacitidine in treatment-naive, elderly patients with acute myeloid leukemia. Blood (2019) 133(1):7–17. doi: 10.1182/blood-2018-08-868752 30361262 PMC6318429

[22] Ghoneim HE Fan Y Moustaki A Abdelsamed HA Dash P Dogra P. *De Novo* Epigenetic programs inhibit PD-1 blockade-mediated T cell rejuvenation. Cell (2017) 170(1):142–57. doi: 10.1016/j.cell.2017.06.007 PMC5568784 28648661

[23] Lee PP Fitzpatrick DR Beard C Jessup HK Lehar S Makar KW. A critical role for Dnmt1 and DNA methylation in T cell development, function, and survival. Immun (Cambridge Mass.) (2001) 15(5):763–74. doi: 10.1016/S1074-7613(01)00227-8 11728338

[24] Craig Chappell CBJA. DNA Methylation by DNA methyltransferase 1 is critical for e ector CD8 T cell expansion 1. J Immunol (2006) 176(8):4562–72. doi: 10.4049/jimmunol.176.8.4562 16585546

[25] Cherkassky L Morello A Villena-Vargas J Feng Y Dimitrov DS Jones DR. Human CAR T cells with cell-intrinsic PD-1 checkpoint blockade resist tumor-mediated inhibition. J Clin Invest (2016) 126(8):3130–44. doi: 10.1172/JCI83092 PMC4966328 27454297

[26] Youngblood B Hale JS Kissick HT Ahn E Xu X Wieland A. Effector CD8 T cells dedifferentiate into long-lived memory cells. Nature (2017) 552(7685):404–9. doi: 10.1038/nature25144 PMC5965677 29236683

[27] McLellan AD Ali HRS. Chimeric antigen receptor T cell persistence and memory cell formation. Immunol Cell Biol (2019) 97(7):664–74. doi: 10.1111/imcb.12254 31009109

[28] You L Han Q Zhu L Zhu Y Bao C Yang C. Decitabine-mediated epigenetic reprograming enhances anti-leukemia efficacy of CD123-targeted chimeric antigen receptor T-cells. Front Immunol (2020) 11:1787. doi: 10.3389/fimmu.2020.01787 32973749 PMC7461863

[29] Wang Y Tong C Dai H Wu Z Han X Guo Y. Low-dose decitabine priming endows CAR T cells with enhanced and persistent antitumour potential *via* epigenetic reprogramming. Nat Commun (2021) 12(1):409. doi: 10.1038/s41467-020-20696-x PMC7814040 33462245

[30] Stübig T Badbaran A Luetkens T Hildebrandt Y Atanackovic D Binder TMC. 5-azacytidine promotes an inhibitory T-cell phenotype and impairs immune mediated antileukemic activity. Med Inflamm (2014) 2014:1–12. doi: 10.1155/2014/418292 PMC3976863 24757283

[31] Joseph A Fraietta SFLE Mercy Gohil SLAC Wilcox FBCD Minnal Gupta RMYF Sadik H. Kassim MMDB. Determinants of response and resistance to CD19 chimeric antigen receptor (CAR) T cell therapy of chronic lymphocytic leukemia. Nat Med (2018) 24(5):563–71. doi: 10.1038/s41591 PMC6117613 29713085

[32] Ladle BH Li K Phillips MJ Pucsek AB Haile A Powell JD. *De novo* DNA Methylation by DNA methyltransferase 3a controls early effector CD8+ T-cell fate decisions following activation. Proc Natl Acad Sci (2016) 113(38):10631–6. doi: 10.1073/pnas.1524490113 PMC5035851 27582468

[33] DiNardo CD Maiti A Rausch CR Pemmaraju N Naqvi K Daver NG. 10-day decitabine with venetoclax for newly diagnosed intensive chemotherapy ineligible, and relapsed or refractory acute myeloid leukaemia: a single-centre, phase 2 trial. Lancet Haematology (2020) 7(10):e724–36. doi: 10.1016/S2352-3026(20)30210-6 PMC7549397 32896301

[34] Schmelz K Wagner M Dörken B Tamm I. 5-Aza-2′-deoxycytidine induces p21WAF expression by demethylation of p73 leading to p53-independent apoptosis in myeloid leukemia. Int J Cancer (2005) 114(5):683–95. doi: 10.1002/ijc.20797 15609309

[35] Greve G Schüler J Grüning BA Berberich B Stomper J Zimmer D. Decitabine induces gene derepression on monosomic chromosomes : *In Vitro* and *In Vivo* effects in adverse-risk cytogenetics AML. Cancer Res (2021) 81(4):834–46. doi: 10.1158/0008-5472.CAN-20-1430 33203699

[36] Que Y Li H Lin L Zhu X Xiao M Wang Y. Study on the immune escape mechanism of acute myeloid leukemia with DNMT3A mutation. Front Immunol (2021) 12. doi: 10.3389/fimmu.2021.653030 PMC8173207 34093541

