# Supplementary material for: Demethylating therapy increases cytotoxicity of CD44v6 CAR-T cells against acute myeloid leukemia
Source: Front Immunol. 2023 Apr 27;14:1145441. doi: 10.3389/fimmu.2023.1145441 (PMC10174291; doi:10.3389/fimmu.2023.1145441)
Supplement: Supplementary file 1 [file DataSheet_1.docx]

Supplementary Material

**Supplementary figures**


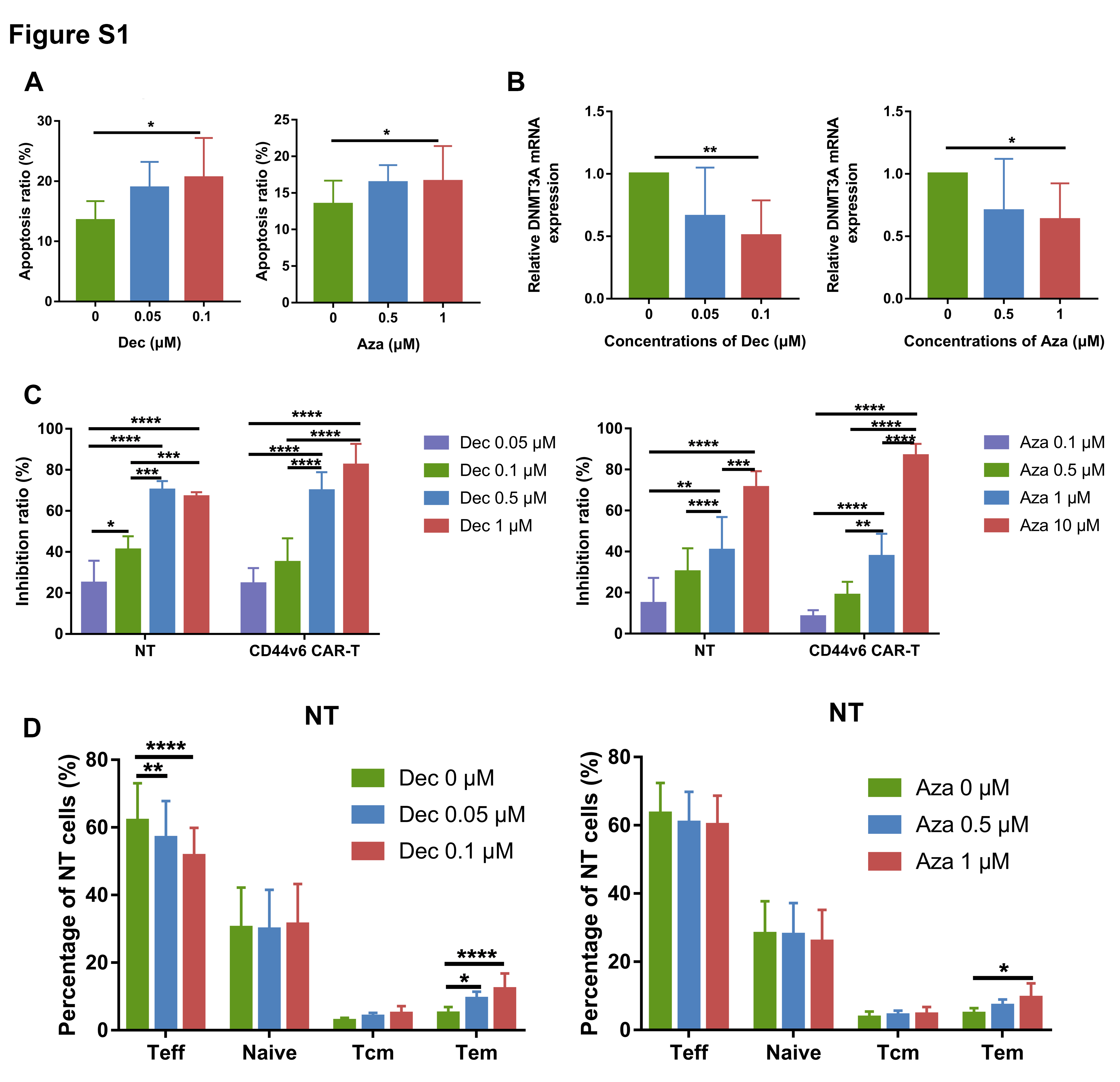


**Figure S1. Effects of Dec and Aza on T cells**

1. Apoptosis of CD44v6 CAR-T cells treated with Dec (0.05, 0.1 μM) (left, n=5) and Aza (0.5, 1 μM) (right, n=5) for 6 days. (B) RT-qPCR analysis of the expression of DNMT3A in CD44v6 CAR-T cells (n=7) treated with Dec (left) and Aza (right) for 6 days. (C) Inhibition ratio of Non-transduced T (NT) cells and CD44v6 CAR-T cells (n≥6) treated with various concentrations of Dec (left) and Aza (right) for 6 days. (D) The changes of phenotype in NT cells (n=6) treated with Dec (left) and Aza (right) for 6 days. Bar are depicted as the mean ± SD. **p*< .05; ***p*< .01; ****p*< .001; *****p*< .0001; ns not significant.


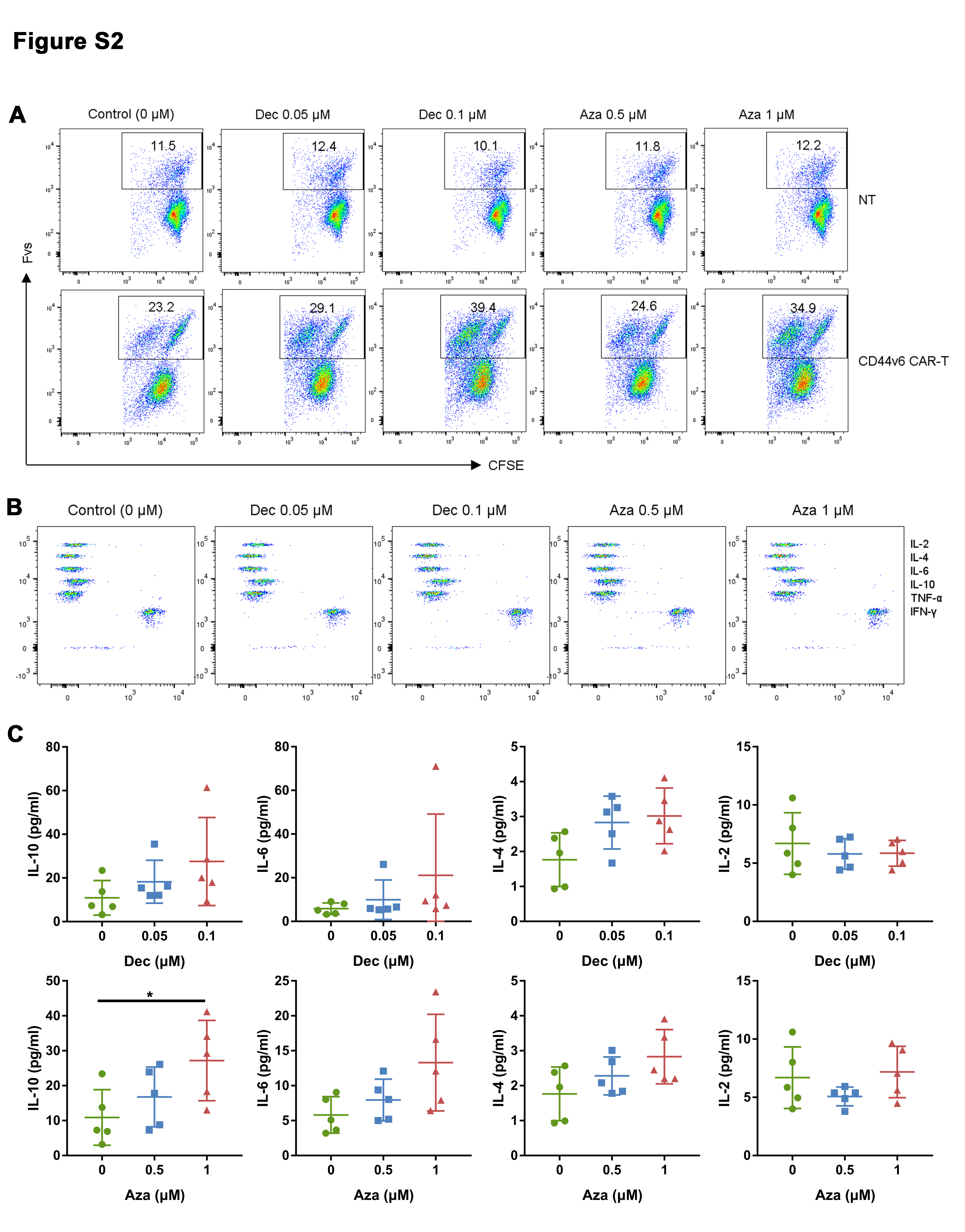


**Figure S2. Dec and Aza pretreated CD44v6 CAR-T cells exhibit enhanced anti-tumor ability towards AML**

1. Representative flow cytometry of the cytotoxicity of Dec and Aza pretreated NT and CD44v6 CAR-T targeting MV4-11 cells. (B-C) Cytokines produced by Dec and Aza treated CD44v6 CAR-T cells co-cultured with MV4-11 cells. Representative flow cytometry is shown in B, and scatter plots of cytokine analysis are shown in C.


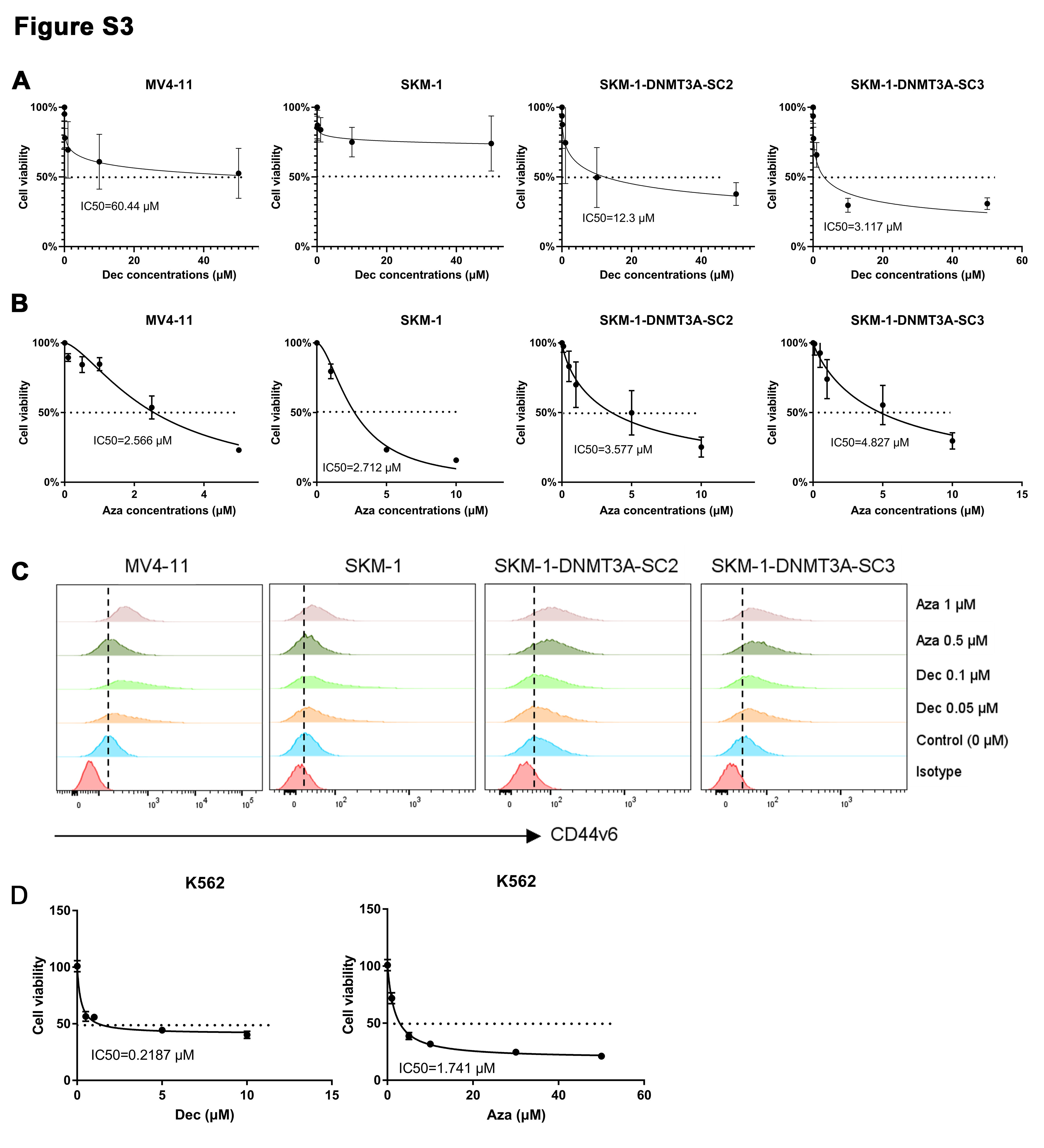


**Figure S3. Effects of Dec and Aza on the proliferation and CD44v6 expression of tumor cells**

(A-B) Effect of Dec (A) and Aza (B) on the proliferation of MV4-11, SKM-1, SKM-1-DNMT3A-SC2 and SKM-1-DNMT3A-SC3 cells and the IC50 of these cells to Dec and Aza. The results were analysed using nonlinear regression curve fit. (C) Representative flow cytometry of CD44v6 expression in MV4-11, SKM-1, SKM-1-DNMT3A-SC2, SKM-1-DNMT3A-SC3 cells treated with Dec and Aza for 3 days. (D) Effect of Dec (left) and Aza (right) on the proliferation of K562 cells and the IC50 of K562 cells to Dec and Aza.
